# HGCS-Det: A Deep Learning-Based Solution for Localizing and Recognizing Household Garbage in Complex Scenarios

**DOI:** 10.3390/s25123726

**Published:** 2025-06-14

**Authors:** Houkui Zhou, Chang Chen, Zhongyi Xia, Qifeng Ding, Qinqin Liao, Qun Wang, Huimin Yu, Haoji Hu, Guangqun Zhang, Junguo Hu, Tao He

**Affiliations:** 1 College of Mathematics and Computer Science, Zhejiang A & F University, Hangzhou 311300, China; 2 Zhejiang Provincial Key Laboratory of Forestry Intelligent Monitoring and Information Technology, Hangzhou 311300, China; 3 College of Information Science and Technology, Zhejiang Shuren University, Hangzhou 311300, China; 4 College of Information Science and Electronic Engineering, Zhejiang University, Hangzhou 310027, China; 5 State Key Laboratory of CAD & CG, Hangzhou 310027, China

**Keywords:** garbage detection, normalization attention, attention-feature fusion, instance boundary reinforcement, Slide Loss

## Abstract

With the rise of deep learning technology, intelligent garbage detection provides a new idea for garbage classification management. However, due to the interference of complex environments, coupled with the influence of the irregular features of garbage, garbage detection in complex scenarios still faces significant challenges. Moreover, some of the existing research suffer from shortcomings in either their precision or real-time performance, particularly when applied to complex garbage detection scenarios. Therefore, this paper proposes a model based on YOLOv8, namely HGCS-Det, for detecting garbage in complex scenarios. The HGCS-Det model is designed as follows: Firstly, the normalization attention module is introduced to calibrate the model’s attention to targets and to suppress the environmental noise interference information. Additionally, to weigh the attention-feature contributions, an Attention Feature Fusion module is employed to complement the attention weights of each channel. Subsequently, an Instance Boundary Reinforcement module is established to capture the fine-grained features of garbage by combining strong gradient information with semantic information. Finally, the Slide Loss function is applied to dynamically weight hard samples arising from the complex detection environments to improve the recognition accuracy of hard samples. With only a slight increase in parameters (3.02M), HGCS-Det achieves a 93.6% mean average precision (mAP) and 86 FPS on the public HGI30 dataset, which is a 3.33% higher mAP value than from YOLOv12, and outperforms the state-of-the-art (SOTA) methods in both efficiency and applicability. Notably, HGCS-Det maintains a lightweight architecture while enhancing the detection accuracy, enabling real-time performance even in resource-constrained environments. These characteristics significantly improve its practical applicability, making the model well suited for deployment in embedded devices and real-world garbage classification systems. This method can serve as a valuable technical reference for the engineering application of garbage classification.

## 1. Introduction

In recent years, with the acceleration of industrialization, the rise in people’s consumption levels and the diversification of consumption structures, the amount of urban domestic garbage has shown a linear upward trend. According to a report from the World Bank, it is anticipated that after 2050, the annual production of garbage will exceed 3.4 billion tons [1]. Consequently, how to curb the escalating volume of garbage has become a serious global social issue. As widely recognized, the fundamental solution to the problem of urban garbage lies in “reduction”, “harmlessness”, and “resourcefulness”, and garbage classification is a necessary prerequisite and a key initiative in realizing a circular economy [2]. However, there are certain shortcomings in the current management of garbage separation. On the one hand, due to shallow subjective awareness or the lack of mandatory constraints from the government, residents’ willingness to participate in garbage classification is not robust, thus increasing the difficulty of garbage treatment and classification [3]. On the other hand, the current work of garbage treatment and classification predominantly relies on manual labor. This method suffers from issues such as low sorting efficiency, high labor intensity and poor hygiene conditions, which pose risks to the physical and mental health of workers and affect the normal operation of garbage sorting management. Therefore, how to guide people to correctly classify garbage and how to change the current garbage classification work mode is particularly important. With the rapid developments in artificial intelligence technology, intelligent classification provides a new effective solution to the current problem of garbage classification.

In recent years, object detection technology has emerged as a focal point in the field of computer vision. Due to its ability to recognize and locate targets in images or videos, it has found widespread application in engineering practices. Similarly, applying object detection technology to garbage classification can significantly enhance the classification efficiency. Traditional object detection algorithms extract target features through feature operators, which are represented by scale-invariant feature transformation (SIFT) [4], histogram of orientation gradients (HOG) [5], Harr-like features [6], etc. Viola et al. [7] utilized Haar-like wavelet features and integral map computation for face detection based on the AdaBoost algorithm. Dalal et al. [5] employed HOG to extract image target features and used SVM to classify pedestrians. For garbage detection, Salimi et al. [8] utilized the Haar-Cascade method to initially detect ground garbage and then combined this with a gray-level co-occurrence matrix and HOG to analyze the garbage features in terms of texture and shape, feeding the results into an SVM for classification. The above detection methods focus solely on low-level feature extraction (e.g., color, texture, shape) of targets in a specific scene, with poor robustness. And they suffer from issues such as low detection accuracy and slow processing speed, thus limiting their application scope. With the rise of deep learning technology, convolutional neural networks (CNNs) break through the technical bottleneck problem of traditional object detection algorithms in feature extraction, and their powerful hierarchical representation ability shines in all kinds of detection tasks. Currently, deep learning-based object detection algorithms can be broadly categorized into two main types: one-stage detection algorithms, represented by the YOLO series [9,10,11] and SSD [12], and two-stage detection algorithms, represented by the R-CNN series [13,14] and SPP-Net [15]. The former directly decode and calculate the output detection result from images, providing higher detection efficiency. The latter first extract candidate bounding boxes from images and then perform secondary correction on the candidate regions to obtain accurate classification and detection results. However, they tend to exhibit lower real-time performance.

In this work, the term “complex environments” refers to real-world conditions in which garbage images are captured, with high variability in backgrounds, illumination, angles, object occlusions, and scale. Such environments introduce noise and ambiguity, making it difficult for models to distinguish foreground garbage from the surroundings and increasing the likelihood of misclassification. In complex garbage detection scenarios, existing models face several critical challenges that hinder their performance and practical deployment. Firstly, they often suffer from attention bias, struggling to consistently focus on relevant object features in cluttered and dynamic environments. Secondly, the irregular and diverse characteristics of garbage objects—such as varying shapes, textures, and sizes—complicate effective feature extraction and accurate boundary localization. Thirdly, sample imbalance arises from the frequent occurrence of hard samples (e.g., low-contrast or partially occluded objects), which are typically underrepresented during training, thereby reducing the model’s generalization ability and detection accuracy.

In response to the challenges of garbage detection in complex scenarios, this paper proposes an improved deep learning solution, namely HGCS-Det. The main contributions of this work are as follows:

1. Enhanced Attention Calibration: We introduce a normalization-based attention mechanism to guide the model toward critical target features while suppressing background noise. Furthermore, an Attention Feature Fusion (AFF) module is designed to adaptively integrate attention weights across channels using instance normalization and channel shuffling, ensuring more effective utilization of attention features.
2. Instance Boundary Reinforcement: To improve the extraction of fine-grained features from irregularly shaped garbage objects, we propose an Instance Boundary Reinforcement (IBR) module. This module fuses gradient-based boundary cues with high-level semantic features to strengthen the representation of object contours.
3. Hard-Sample Optimization with Slide Loss: We incorporate the Slide Loss function to dynamically reweight hard samples during training. This strategy improves the model’s sensitivity to ambiguous samples in transitional regions between the background and foreground, enhancing the overall detection accuracy.

## 2. Related Work

In recent years, growing interest in lightweight and efficient garbage detection networks has led to the emergence of several promising architectures. For instance, Chen et al. proposed LWCNet [16], a YOLOv8n-based model that integrates a self-attention detection head (SAHead) for enhanced contextual modeling, GSConv for parameter-efficient convolution, and a GRCSPELAN block that combines GSConv with GELAN to reduce model complexity without sacrificing accuracy. They also introduced the AIFI module to strengthen intra-scale feature interactions. LWCNet demonstrates notable improvements in inference speed and compactness, making it suitable for deployment on edge devices. However, its performance in highly complex scenes with occlusions or irregular objects remains underexplored. Similarly, Wang et al. developed a mobile-cart-deployed variant of YOLOv8n for small-object detection [17]. Their model incorporates a C2f-MS backbone to preserve multi-scale details while reducing the parameter count. Additionally, a novel Convergence–Expansion Pyramid Network (CEPN) was proposed to improve semantic feature flow in the neck, and a QS-Dot-IoU hybrid loss was designed to jointly optimize classification and localization. This architecture achieved strong performance on small-target datasets with limited computational cost. Nevertheless, its generalization ability across diverse environments and garbage types has not been extensively validated. Meanwhile, CNN-based object detectors have achieved encouraging results in garbage classification tasks, significantly advancing the development of intelligent waste management. For example, Chen et al. [18] redesigned YOLOv5s using ShuffleNetv2 and depthwise separable convolutions, achieving a 94% mAP with only 5.3M parameters and faster inference on Jetson Nano. Despite its speed, the model’s detection robustness in real-world noisy scenes remains limited. In another study, Sun et al. [19] introduced SRAF-Net, a shape-robust anchor-free network designed for remote sensing garbage detection. It combined contextual attention with deformable convolution to focus on subtle features and adopted Merge NMS to address boundary blurring. While effective in remote sensing scenarios, its performance in close-range or densely cluttered environments was not discussed. Lee et al. [20] designed a practical automatic garbage collection system combining a pruned SSD model with a robotic arm. The system achieved 88.5% accuracy at 13–16 FPS, but its detection precision is moderate and may not scale well to complex, multi-class settings. Research focus has also expanded to multi-target garbage detection. Mao et al. [21] compared YOLOv3 trained on single-target TrashNet and multi-target TRWD datasets, revealing superior performance on dense garbage images with the latter. Zhang et al. [22] established the MULTI-TRASH dataset and applied transfer learning with YOLOv4, achieving a 93.12% mAP. However, these models are often trained in constrained, single-domain datasets, limiting their adaptability when deployed across different environmental contexts. To overcome single-scene constraints, Majchrowska et al. [23] aggregated several public datasets to build Detect-Waste, a benchmark covering indoor, outdoor, and underwater garbage. Models such as EfficientDet, DETR, and Mask R-CNN were evaluated, with EfficientDet showing the best overall performance. Nonetheless, the employed models were computationally heavy, limiting their practical deployment on embedded or mobile platforms. Addressing the multi-scenario challenge, Lun et al. [24] proposed Skip-YOLO, based on YOLOv3, with densely connected blocks for feature fusion. Although it achieved a 90.38% mAP, the dense architecture substantially increased the computational load, compromising real-time performance. Li et al. [25] proposed an attention-augmented Faster-RCNN model for unmanned surface vehicles (USVs) to detect floating waste. While it achieved satisfactory results, the method suffered from slow inference and a reliance on complex post-processing steps, making it less feasible for embedded systems.

In summary, while recent studies have explored various improvements—ranging from lightweight backbones and enhanced attention mechanisms to multi-scale detection and hybrid loss functions—most existing models face limitations when applied to real-world complex environments. These include large model sizes, reduced adaptability to varying backgrounds and object shapes, and insufficient performance on hard samples such as occluded or overlapping waste. These gaps highlight the need for a solution that achieves a better trade-off between accuracy, speed, and generalization in complex scenarios—a goal that this paper aims to address with the proposed HGCS-Det model.

## 3. Materials and Methods

### 3.1. Dataset HGI30

This study evaluates the performance of the proposed model using the publicly available household garbage dataset HGI30 [26]. HGI30 totals 18,178 garbage images containing 30 common types of household garbage distributed across several complex natural scenarios. The images in HGI30 were collected using a variety of commercial off-the-shelf imaging devices, including smartphones and consumer digital cameras. These devices are equipped with typical CMOS image sensors, which are widely used in embedded and mobile imaging systems. The sensor resolutions of the devices range from approximately 8 MP to 48 MP, depending on the model and manufacturer, resulting in image resolutions varying from 300 × 400 to 3000 × 4000 pixels. Data were captured under natural lighting conditions, without the use of artificial lighting or controlled environments, to better reflect the challenges of real-world garbage detection tasks.

**Figure 1:**
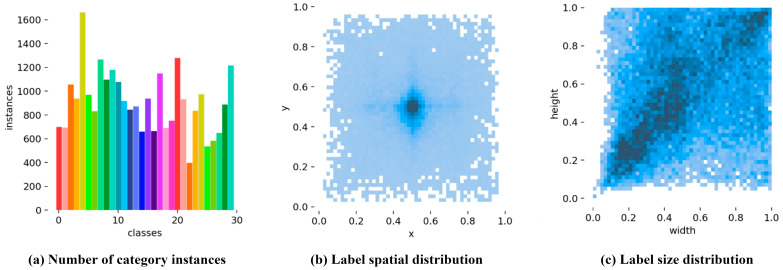
Dataset analysis for (**a**) the number of category instances, (**b**) the label spatial distribution, and (**c**) the label size distribution. (**a**) This bar chart displays the number of samples for each category across 30 classes. Different colors are used solely to distinguish each category visually, enhancing clarity and making it easier to identify individual class distributions. (**b**) This heatmap represents the spatial distribution of labels within the dataset. The intensity of the color indicates density, with darker blue shades signifying higher concentrations of data points. (**c**) This heatmap illustrates the distribution of label sizes (height vs. width). Similar to (**b**), darker blue shades represent higher densities, with a pronounced concentration in the upper right quadrant, indicating that larger label sizes are more prevalent in the dataset.

**Figure 2:**
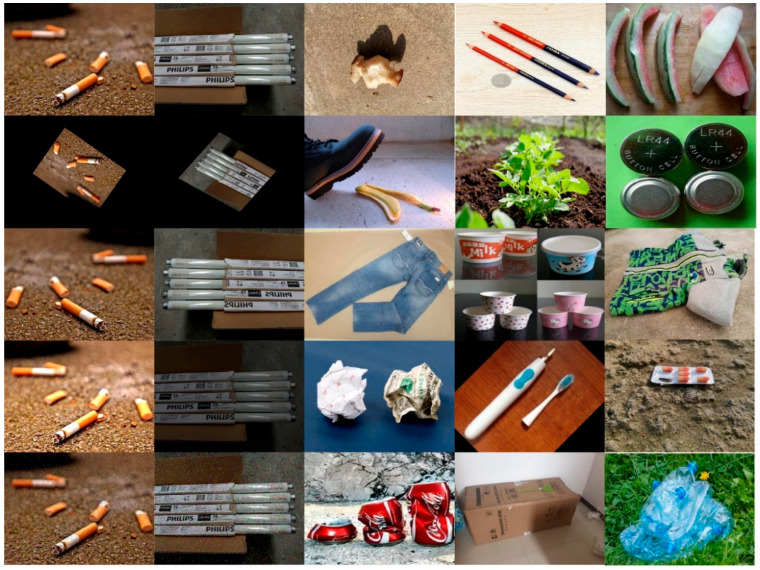
Examples of data diversity in HGI30.

The dataset captures both fixed features and deformable features (shape and texture) of each type of garbage to cope with challenges arising from intra-class variations and inter-class similarities. Additionally, data augmentation techniques such as rotation, flipping, noise addition, and brightness adjustments are applied to enrich the diversity of garbage features. Furthermore, the images’ resolutions range from 300 × 400 to 3000 × 4000. Each image contains one or more garbage instance targets, accommodating single-target and multi-target garbage detection scenarios under diverse scales. The number of instances per category in the HGI30 dataset is relatively balanced, with only a few low-frequency categories (Figure 1). The targets in the dataset are dispersed from the center of the images toward the edges, exhibiting no significant spatial bias. Additionally, the target positions are diverse, and the width–height ratios of the objects cover a wide range, indicating a well-distributed and representative dataset. In summary, the garbage images in HGI30 exhibit characteristics such as complex backgrounds, variable lighting conditions, diverse angles, varied shapes, and multi-scale variations. The dataset’s diversity helps alleviate overfitting issues, strengthening the model’s generalization performance. This dataset establishes a benchmark for subsequent model improvements. Examples of data diversity in HGI30 are illustrated in the images shown in Figure 2. For subsequent experiments, the dataset is randomly split into the training set and the test set according to the ratio of 8:2.

### 3.2. Overview of YOLOv8

**Figure 3:**
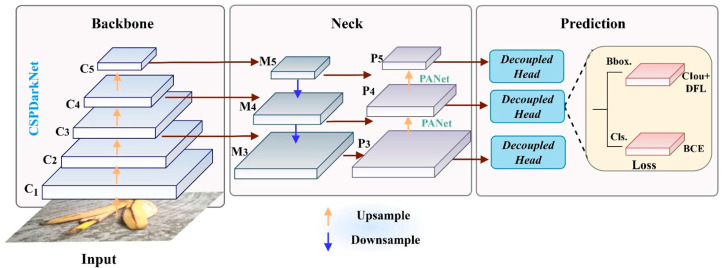
The overall architecture of YOLOv8.

The YOLOv8 model [27] is the latest version of the YOLO series, and it offers heightened flexibility and superior detection performance, thus rendering it more applicable to detection tasks in practical projects. In terms of its operational principle, YOLOv8 employs an anchor-free paradigm to directly generate high-quality bounding boxes, and in conjunction with the dynamic label-assignment strategy Task-Aligned Assigner (TAL), matches appropriate positive samples for different targets. This simplifies the network construction and training, enhancing the model’s generalization capabilities. The architecture of YOLOv8 consists of four parts: Input, Backbone, Neck, and Prediction (Figure 3).

**Input.** The Input component primarily preprocesses input images through the data augmentation method Mosaic and adaptive image scaling. In the Mosaic method, four images are stitched together through random scaling, cropping, and sorting, allowing the model to learn rich background information from diverse image data and enhancing its robustness.

**Backbone.** The Backbone continues the design philosophy of the traditional CSPDarkNet53 [28], with the only modification being the replacement of the C3 module with the C2f module, which possesses stronger gradient flow information. The C2f module, inspired by the Extended Efficient Layer Aggregation (ELAN) module design of the YOLOv7 model [29], enables the learning of richer gradient combination information by cross-layer concatenation aggregation across branches, thus enhancing the feature-extraction capability.

**Neck.** The Neck employs the combined structure of a Feature Pyramid Structure (FPN) and a Path Aggregation Network (PAN). It acquires multi-level, high-dimensional semantic information by propagating features in a top–down and a bottom–up manner.

**Prediction.** The Prediction component introduces three decoupled heads of different scales. Through two independently parallel sub-networks, it obtains precise localization coordinates and classification scores for targets of different scales, achieving accurate positioning and recognition. Unlike other detectors, YOLOv8’s loss calculation excludes the objectness-loss branch, only including the classification-loss branch using the BCEWithLogitsLoss function and the localization regression-loss branch using the combined functions of DFL Loss and CIoU Loss. The total loss is represented as follows:

(1) $$L=\alpha L_{CIou}+\beta L_{DFL}+\gamma L_{BCE}$$

where α, β, and γ are the loss–gain factors for the training setup.

### 3.3. The Proposed Model

**Figure 4:**
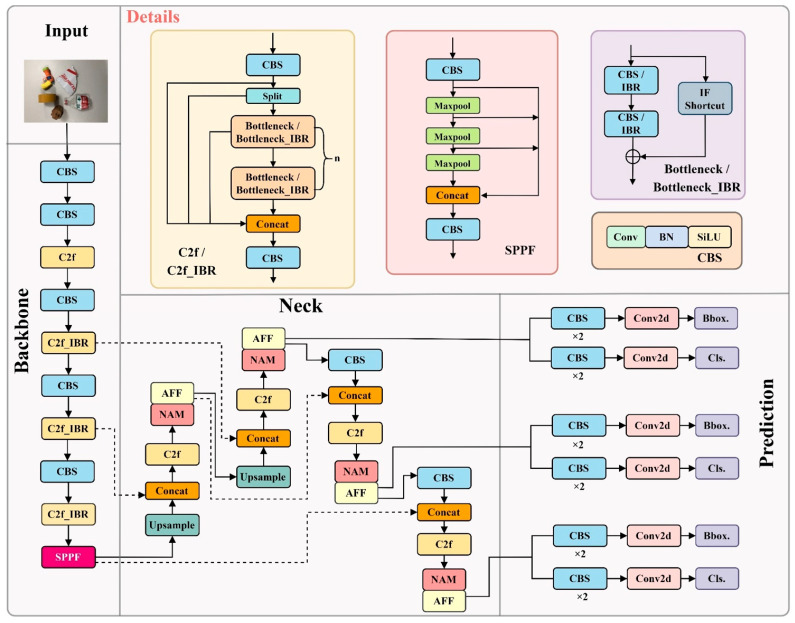
The structure of the HGCS-Det model. Solid arrows represent the main data flow, highlighting the direct transmission of feature maps across the Backbone, Neck, and Prediction stages. In contrast, dashed arrows signify residual or skip connections, whereby features from preceding layers are concatenated or summed with subsequent layers to retain spatial details.

The garbage detection task requires high accuracy in real-time and embeddability as performance support. Therefore, this paper selects the flexible and lightweight YOLOv8n as the baseline model for improvement and proposes the HGCS-Det model based on this. The structure of the HGCS-Det model is illustrated in Figure 4. Firstly, the attention mechanism NAM [30] is integrated after each C2f module in the Neck to enhance the model’s focus on informative features—such as object contours and texture patterns—within deep semantic representations while effectively suppressing irrelevant or redundant background noise. Meanwhile, in conjunction with the Attention Feature Fusion (AFF) module, the weights of attention features from different channels are integrated to enhance the model’s attention to targets and to obtain the fine-grained features of garbage, facilitating the distinction of features when imposed on the feature-extraction layer of the backbone. Specifically, convolutions in the Bottleneck are replaced with the IBR module, forming the C2f_IBR module. C2f_IBR enhances the model’s modeling capability by fusing rich boundary information with gradient flow combination information. Finally, the Slide weight function with an adaptive threshold is introduced to make the model pay more attention to hard samples during training. The above four improvement methods will be described in detail in the following sections.

### 3.4. Normalization-Based Attention Module

CNNs suffer from the indiscriminate nature of convolutional feature encoding, leading to dispersed attention to targets and the generation of useless feature information. In addition, garbage detection tasks in natural scenarios are frequently plagued by background and lighting. Therefore, there is a greater need for a method to suppress noisy information and to focus on the targets’ key features. Attention mechanisms enable the capture of crucial information from vast amounts of data, which is beneficial for coping with detection tasks in complex scenarios [25].

**Figure 5:**
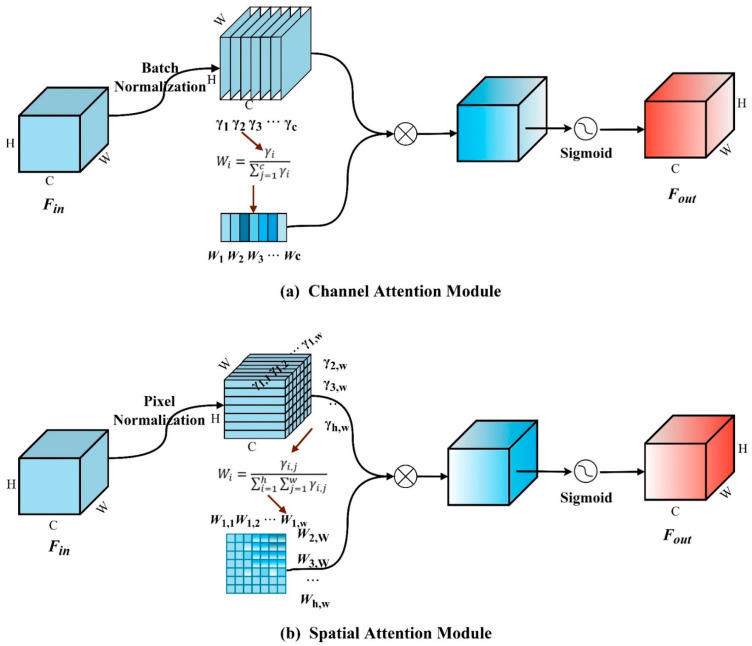
The structure of NAM, which is made up of the combination of (**a**) a channel attention module and (**b**) a spatial attention module.

This study introduces a Normalization-based Attention Module (NAM) to calibrate the model’s attention. The NAM [30] is a normalization-based attention mechanism that selects the dimension weight contribution value as a measure of the salient features in images. Specifically, the NAM assesses the importance of each dimension feature weight through the scaling factor of batch normalization, suppressing unimportant channel or pixel information in the image and enhancing the target feature representation. Similar to the integration method of the attention mechanism CBAM [31], the NAM consists of two sub-modules: channel attention and spatial attention (Figure 5).

**Channel attention module.** This module utilizes a sparsity penalty on channel weights to calibrate the channel information of the feature map. For an input feature $F_{in}\in R^{H\times W\times C}$ with pixel dimension H∗W and channel dimension C, the process begins with batch normalization (BN) to calculate and extract the scaling factor γ of BN; then, the importance ratio of the scaling factor $\gamma_{i}$ for each channel is calculated as the channel weight contribution value $W_{i}$; next, the channel feature information are recalibrated through the matrix multiplication of $W_{i}$ with the normalized feature; finally, the attention weight coefficients are computed using the activation function Sigmoid and multiplied with the input features, thus obtaining the final attention-feature map $F_{out}\in R^{H\times W\times C}$. The calculations for the normalization and weight value are expressed as Equations (2) and (3), respectively.

(2) $$BN=\gamma_{B}\frac{F_{in}-\mu_{B}}{\sqrt{\sigma_{B}^{2}+\epsilon }}+\beta_{B}\;$$

(3) $$W_{i}=\frac{\gamma_{i}}{\sum_{j=1}^{C}\gamma_{j}}\;$$

where $B_{F}$ denotes the input feature of the BN layer; $\mu_{B}$ and $\sigma_{B}$ denote the mean and variance of the input data; and $\gamma_{B}$ and $\beta_{B}$ denote the scaling and shifting parameters used for learning the channel feature distribution, respectively.

**Spatial attention module.** This module utilizes a sparsity penalty on spatial weights to calibrate the spatial information of the feature map. Its operational principles are similar to the aforementioned channel attention module. The distinction lies in the fact that this module, through dimensional transformation, maps the dimensional information calculated by batch normalization to spatial pixels, i.e., a pixel normalization operation. The normalization and weight value calculations in this module are expressed as Equations (4) and (5), respectively.

(4) $$PN=\gamma_{p}\frac{F_{in}-\mu_{P}}{\sqrt{\sigma_{P}^{2}+\epsilon }}+\beta_{p}\;$$

(5) $$W_{i,j}=\frac{\gamma_{i,j}}{\sum_{i=1}^{H}\sum_{j=1}^{W}\gamma_{i,j}}\;$$

where $\mu_{P}$ and $\sigma_{P}$ denote the mean and variance of the input data, and $\gamma_{p}$ and $\beta_{p}$ denote the scaling and shifting parameters for learning the pixel feature distribution.

### 3.5. Attention Feature Fusion Module

Attention features of each channel generated by the attention mechanism contribute differently to the model. Generally, the model tends to focus on attention features with higher contributions and ignores those with lower weights. This situation results in the model not fully leveraging all the attention features in strengthening the attention to targets. To compensate for the shortcomings caused by polarized attention features, we designed an Attention Feature Fusion (AFF) module to synthesize the weight contributions of all attention features.

**Figure 6:**
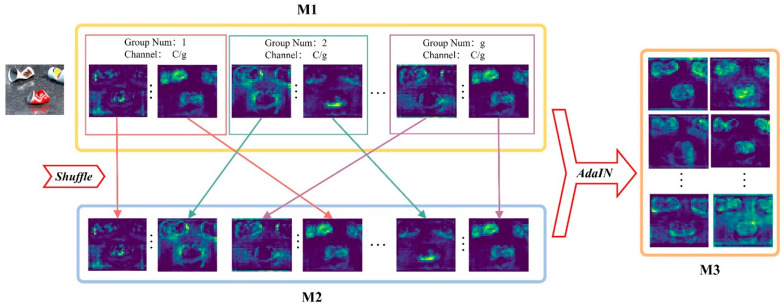
Visualization demonstration of the AFF module principle. These color-coded arrows illustrate the continuous processing stages of the same image across the multi-group feature pipeline.

AFF is a parameter-free attention enhancement module. The working principle of AFF is straightforward, which is implemented employing only two simple operators: Channel Shuffle and Adaptive Instance Normalization (AdaIN) [32]. The feature visualization intuitively demonstrates the specific workflow and effect of AFF (Figure 6). Specifically, for the input attention feature M1, firstly, each channel feature in M1 is rearranged and mixed using the Channel Shuffle operation, reshaping it into another set of attention features, denoted as M2. M2 can be expressed as

(6) $$M2=shuffle\left(M1\left(g,c\right)\right)$$

where g denotes the number of groups for channel splitting, and c denotes the channel dimensions of each group. Next, the AdaIN operator is employed to migrate the styles of M2 to the content features of M1, fusing the attention features with different weight values in the corresponding channels of M1 and M2, resulting in the output feature M3, which significantly highlights the attention to targets. The calculation of M3 is as follows:

(7) $$M3=AdaIN\left(M1,M2\right)=\sigma \left(M2\right)\left(\frac{M1-\mu \left(M1\right)}{\sigma \left(M1\right)}\right)+\mu \left(M2\right)$$

where σ and μ denote the variance and mean of a specific feature.

The AFF module aims to enhance the saliency of attentional features, further improving the model’s robust performance. As a plug-and-play module, AFF is easily applicable after the attention layer. We refer to this combination as the Enhanced Attention Mechanism Module. In this study, we combine AFF with the aforementioned NAM.

### 3.6. Instance Boundary Reinforcement Module

Lightweight detectors are often constrained by their limited representation, resulting in blurred instance boundary features of target objects. Additionally, influenced by complex natural environments, garbage detection models may also struggle to distinguish between foregrounds and backgrounds during training. Such ambiguous instance boundary features could increase the risk of a model’s misclassification. Therefore, in garbage detection tasks, enhancing the boundary information (shape, texture) representation of irregular garbage targets is crucial.

**Figure 7:**
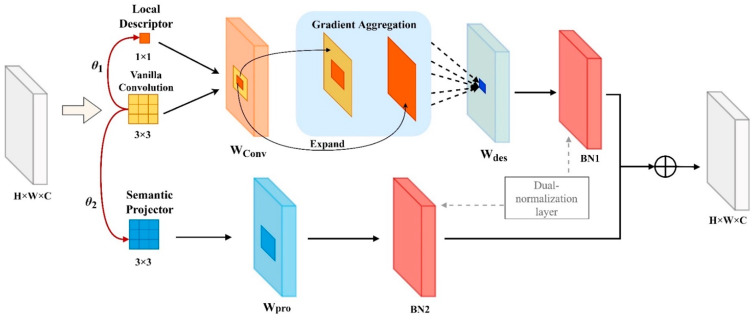
The structure of the IBR module. Solid red arrows denote the primary data flow, guiding the input through the Local Descriptor and Semantic Projector, where convolutions extract initial features, directing the process to subsequent modules for further refinement. Dashed black arrows illustrate the gradient aggregation and expansion within the Gradient Aggregation module, demonstrating how gradients are collected and expanded to enhance feature quality before advancing to the next stage.

In recent research, the Instance Boundary Enhancement (IBE) Module [33] captures target boundary information by integrating gradient cues from a shared depth-separable convolution (DSC) around a difference convolution, aiming to ameliorate the low-performance issues caused by DSC. However, due to the independence of DSC’s computation, the lack of interaction between channel information may result in the loss of target detail information, ultimately affecting model performance. Therefore, this study, which is focused on garbage detection tasks, proposes the Instance Boundary Reinforcement (IBR) Module, building upon the IBE Module. We set the shared convolution as a more interactive vanilla convolution. Simultaneously, we eliminate the point convolution in the output layer of IBE to reduce the computational costs. The IBR module mainly consists of a shared convolution, a local descriptor, a semantic projector, and a dual-normalization layer (Figure 7). The local descriptor employs a gradient difference mechanism, i.e., central difference convolution, generated through gradient aggregation from the shared convolution. The semantic projector derives feature semantic information by learning the feature mapping of the shared convolution. The dual-normalization layer comprises two independent batch normalization layers, aligning the output features.

Specifically, given an input feature x, the local receptive field region of x is first sampled by a 3 × 3 vanilla convolution, followed by aggregation of the weighted sum of the sampled values to produce the shared feature weight matrix $W_{Conv}$. The calculation of $W_{Conv}$ is as follows:

(8) $$W_{Conv}=\sum_{P_{n}\in R}w\left(P_{n}\right)\cdot x\left(P_{n}\right)\;$$

where R denotes the local receptive field region for the convolution operation, w denotes the filter used to sample the features, and $P_{n}$ denotes the weight value at the *n-th* position in R. Next, the feature information of $W_{Conv}$ is focused on the central position of the region R. To obtain the central gradient information, the weight value located at the central position is set to 0. The feature $W_{des}$ with its rich boundary information is obtained through gradient-difference computation.

The calculation of $W_{Conv}$ is as follows:

(9) $$\begin{array}{c} W_{des}=\theta_{1}\sum_{P_{n}\in R}w\left(P_{n}\right)\cdot \left(x\left(P_{n}+P_{0}\right)-x\left(P_{0}\right)\right)+\left(1-\theta_{1}\right)\sum_{P_{n}\in R}w\left(P_{n}\right)\cdot x\left(P_{n}\right)\\ =\sum_{P_{n}\in R}w\left(P_{n}\right)\cdot x\left(P_{n}\right)-\theta_{1}\cdot x\left(P_{0}\right)\sum_{P_{n}\in R}w\left(P_{n}\right)\;\# \end{array}$$

where $P_{0}$ denotes the weight value at the central position of the local receptive field region, and $\theta_{1}\in [0,\;1]$ is a learnable parameter used to measure the importance of the gradient information. Meanwhile, to compensate for the low semantic information due to gradient features in the difference convolution, the feature $W_{Conv}$ is used to learn the generalized abstract semantic information $W_{pro}$. The calculation of $W_{pro}$ is as follows:

(10) $$W_{pro}=\theta_{2}\sum_{P_{n}\in R}w\left(P_{n}\right)\cdot x\left(P_{n}\right)$$

where $\theta_{2}\in [0,\;1]$ is a learnable parameter used to learn the importance of the semantic projection. Finally, the features of $W_{des}$ and $W_{pro}$ are aligned using dual-normalization (${BN}_{1}$, ${BN}_{2}$) and summed, thus obtaining the output feature $X_{out}$. $X_{out}$ can be expressed as

(11) $$X_{out}={BN}_{1}\left(W_{des}\right)+{BN}_{2}\left(W_{pro}\right)\;$$

### 3.7. Slide Loss

In one-stage detectors, many bounding boxes are not repeatedly filtered, and there are numerous negative samples with no overlapping region with real objects or positive samples with a high degree of overlap with real objects. These samples are referred to as easy samples. Garbage detection models are often affected by environmental factors and characteristics of the garbage itself. During training, they may generate bounding boxes with unclear classifications, i.e., hard samples, leading to less-than-ideal detection results. Although simple samples have a small individual loss, their large quantity results in a significantly higher loss compared with hard samples. Consequently, the accumulated loss from easy samples dominates model updates, leading to overfitting situations. This represents the imbalance between hard samples and easy samples. Therefore, this study introduces the Slide Loss function with an adaptive threshold to optimize the learning of hard samples.

**Figure 8:**
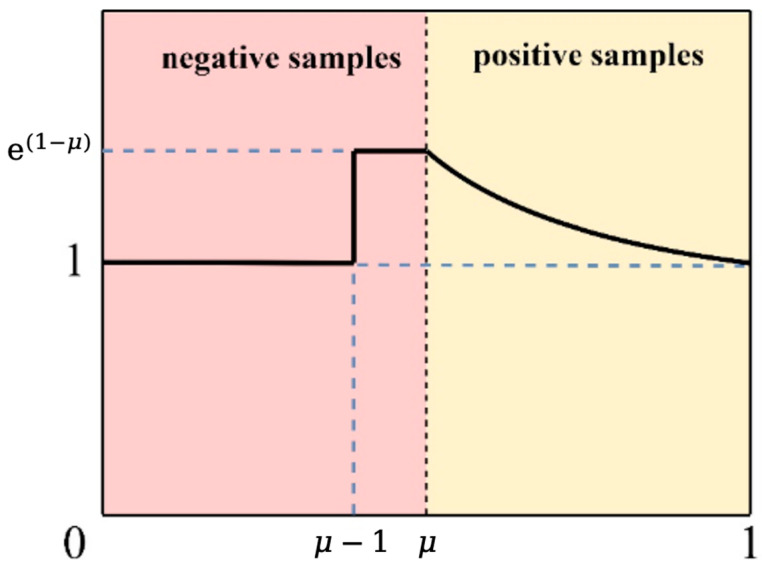
The weighting strategy for Slide Loss [32].

The Slide Loss function [34] is an optimization strategy for positive and negative sample weight allocation introduced in the classification function BCEWithLogitsLoss. During the training process, it achieves dynamic weight allocation by adaptively learning a threshold parameter. As is well known, the distinction between easy samples and hard samples lies in the Intersection Over Union (IoU) values of the predicted and true boxes. Hard samples have bounding boxes with unclear classifications, positioned in the transitional region between the foreground and the background. Although the loss from difficult samples is large, they are relatively sparse in number and are not easily attended to by the model during training. Therefore, higher learning weights need to be assigned to hard samples. The Slide Loss method calculates the average IoU value of all bounding boxes as the threshold μ (Figure 8). Bounding boxes smaller than μ are treated as negative samples, while those larger than μ are treated as positive samples. The region around the threshold is defined as the region prone to generating hard samples. The hard samples are then weighted by the weighting function Slide. The formula for the weighting function Slide is as follows:

(12) $$f\left(x\right)=\left\{\begin{array}{l} 1,\;x\leq \mu -0.1\\ e^{1-\mu },\;\mu <x<\mu -0.1\\ e^{1-x},\;x\geq \mu \end{array}\right.$$

Our code will be available on https://github.com/xzhyi/HGCS-Det (accessed on 26 May 2025).

## 4. Experiments and Results

### 4.1. Experimental Setup

The relevant experiments covered in this paper were conducted in accordance with the following experimental setup to ensure the fairness of the experimental sessions.

**Experimental environment.** The experiments in this study were trained and tested in a Windows 11 operating system. Server configuration: the CPU is Intel i7-13700K, the GPU is NVIDIA RTX 3090 with a 24 G graphics memory, and the deep learning development environment is composed of PyToch 1.10.0, Python 3.8, and CUDA11.3.

**Parameter settings.** The stochastic gradient descent (SGD) algorithm was chosen as the optimizer, and the initial learning rate, the weight decay, and the momentum were set to 0.01, 0.0005, and 0.937, respectively. Prior to training, the input images’ sizes were normalized to 640 × 640, the batch size was set to 32, and the training epochs were set to 300.

### 4.2. Evaluation Metrics

In this study, the evaluation metrics for model performance include the following: parameters (Params), floating-point operations per second (FLOPs), precision (P), recall (R), mean average precision (mAP), and frames per second (FPS). Among them, Params and FLOPs represent the spatial and temporal complexity of the model, serving as crucial considerations in determining the model’s applicability.

Precision (P) represents the proportion of correctly predicted positive samples to the total predicted positive samples. Recall (R) represents the proportion of correctly predicted positive samples to all actual positive samples. The formulas for P and R are as follows:

(13) $$P=\frac{TP}{TP+FP}$$

(14) $$R=\frac{TP}{TP+FN}$$

where TP (true positive) denotes the number of samples correctly judged as positive; FP (false positive) denotes the number of samples incorrectly judged as positive; and FN (false negative) denotes the number of samples incorrectly judged as negative.

The mAP reflects how well the predicted boxes match the true boxes and whether the target category was correctly predicted. The mAP is determined by P and R and represents the mean of the area of the P-R curve (Accuracy Precision) for all target categories. The formula for mAP is as follows:

(15) $$mAP=\frac{1}{n}\sum_{i=1}^{n}AP_{i}$$

where $AP_{i}$ denotes the area of the *P-R* curve of the i-th category.

The FPS parameter represents the number of image frames the algorithm can process in one unit of time, reflecting the algorithm’s operating speed. The formula for FPS is as follows:

(16) $$FPS=\frac{1}{t}$$

where t denotes the time required for processing an image.

### 4.3. Evaluation of the Enhanced Attention Mechanism Module

The attention mechanism effectively suppresses noise in complex scenarios, enhancing the focus on targets. Therefore, we evaluated the contributions of multiple mainstream attention mechanisms (SE [35], CBAM [31], ECA [36], CA [37], and NAM [30]) to garbage detection in complex scenarios, based on the baseline model YOLOv8n. For the NAM, we further assessed its channel attention, NAM^C*^, and spatial attention, NAMS*, separately. Models introducing attention mechanisms exhibited somewhat improved detection accuracy compared with YOLOv8n (Table 1). This confirms the applicability of the attention mechanism to garbage detection in complex scenarios. Among these, the model introducing CA achieves the highest mAP, reaching 93.2%. The model introducing NAM^C*^ followed closely, with an mAP of 92.9%, outperforming the other attention mechanisms. It is noteworthy that the attention mechanisms require multiplication of the generated attention weights with the original features to calibrate the model’s focus. The complex matrix multiplication involved in this process consumes computational resources, leading to a decrease in detection speed.

Next, we evaluated the effectiveness of the Attention Feature Fusion (AFF) module in adjusting the contribution of the attention weights. As observed in Table 1, the enhanced attention modules formed by combining the attention mechanism with the AFF module increased the mAP of each model by 0.1% to 0.4%. This indicates that the attention mechanisms, after synthesizing the attention weights of each channel through AFF, have greater attention than the original attention mechanisms. Among them, the combination of NAM^C*^ and AFF achieves a 93.2% mAP, almost without additional computational costs, which is only 0.1% lower than the highest mAP, and exhibits faster detection speed compared with other enhanced attention modules. Taking these considerations into account, the combination of NAM^C*^ and AFF was chosen as the improvement strategy in this study.

**Figure 9:**
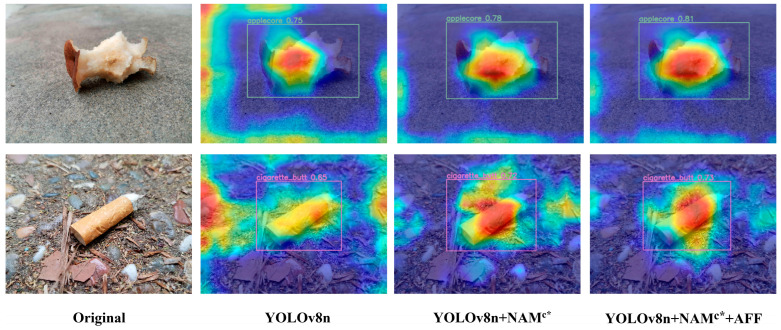
Examples of visual analysis by Grad-CAM. The figure compares detection results for an apple core and a cigarette butt across four columns: the first column shows the original images without overlay, while the subsequent columns display heatmaps where colors transition from blue (low confidence) to red (high confidence), with yellow and orange indicating intermediate levels.

To further validate the effect of this Enhanced Attention Module for calibrating the model’s attention, we employed Grad-CAM for visual analysis in the form of the generation of heatmaps. Grad-CAM [38] maps category activations to the model via inverse gradient computation, allowing us to visually assess the weight size that a given region produces on the predicted output based on the depth of the region’s luminance. The image displays two examples of visualizations generated using Grad-CAM (Figure 9). From the images, due to the equal treatment of features by convolution coupled with the effect of complex scenarios, YOLOv8n’s focus on the target is biased toward environmental noise such as backgrounds, stones, and branches. With the inclusion of NAM^C*^, the model effectively suppresses the influence of noise information, leading to improvements in coverage and attention to the target region. Moreover, the Enhanced Attention Module, incorporating NAM^C*^ and AFF, demonstrates more pronounced global (contour and texture) attention on the garbage targets. The above results indicate that our proposed Enhanced Attention Module can effectively calibrate the model’s focus on the targets, enabling precise localization and identification of garbage objects in complex scenarios. This also indirectly demonstrates the model’s strong generalization performance when applied to complex scenarios.

### 4.4. Evaluation of the Instance Boundary Reinforcement Module

The IBR module is proposed based on the IBE module, which captures the fine-grained features of garbage for discrimination. The table shows the improvement process of the IBR module and its performance evaluation (Table 2). Initially, the IBE module is introduced into the backbone of YOLOv8n, where the model experiences a mere 0.3% decrease in mAP despite a significant reduction in Params and FLOPs. Then, to obtain richer detailed features, the depth-separable convolution (DWC) used for weight sharing in the IBE module is replaced with a vanilla convolution. This modification results in an improved mAP to 92.8% but with an accompanying rise in computational cost, impacting latency. To balance the computational costs and to mitigate the latency loss, the point convolution (PC) used for output features in the IBE module is removed. The model with only the addition of the branch Batch Normalization (BN), incurring almost negligible computational cost, achieves an mAP of 92.7%. Simultaneously, during inference, the reparameterization technique is employed to achieve a detection speed nearly indistinguishable to that from YOLOv8n.

**Figure 10:**
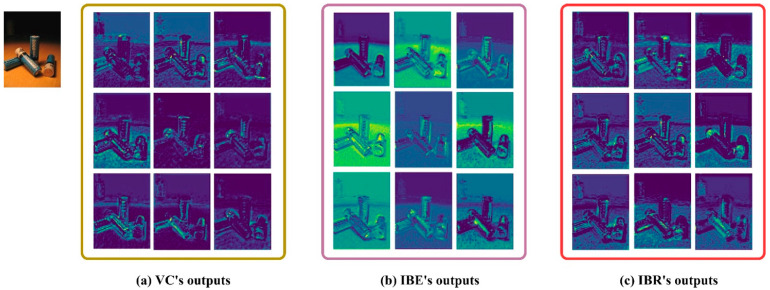
Visual analysis for (**a**) VC’s outputs, (**b**) IBE‘s outputs, and (**c**) IBR’s outputs.

To further validate the superiority of the IBR module in feature representation, we analyzed the visualized features of the vanilla convolution (VC) in the original YOLOv8n and the visualized features replaced with the IBE module and the IBR module. VC, due to its weight interactivity, learns richer feature information, including background information (Figure 10). Consequently, the background in the feature outputs by VC is not obvious, while the target features appear to be somewhat blurred or distorted. The IBE module is able to highlight the main features (outline, shapes, textures) of the garbage targets, providing more feature details, but lacks fitting to the complex background. The IBR module is more like a combination of the advantages of the VC and IBE modules in not only suppressing background information but also in obtaining clear edge features. This demonstrates the superiority of the IBR module in terms of feature expressiveness.

### 4.5. Evaluation of Slide Loss

Slide Loss aims to weight the learning of hard samples, improving the accuracy of such samples. To demonstrate the superiority of Slide Loss in handling hard samples, we evaluated three other loss functions (Focal Loss [39], QFocal Loss [40], and Varifocal Loss [41]) used to address the imbalance problem between easy and hard samples. Focal Loss, QFocal Loss, and Varifocal Loss perform poorly (Table 3). These loss functions require the setting of modulation factors to allocate weights, and due to the uncertainty of these factors, there may be a serious imbalance in the weight distribution between easy and hard samples. Slide Loss can adaptively learn the threshold parameter, achieving dynamic weight allocation. Therefore, Slide Loss achieves an optimal detection result.

To further validate the effectiveness of Slide Loss in improving the recognition accuracy of hard samples, we selected categories validated by YOLOv8n with AP values below 90% and observed whether their AP values could be boosted under the influence of Slide Loss. For most of the classes, their AP values were improved after calibration with Slide Loss (Table 4). Only two categories experienced a decrease in their AP. The reason for this might be because the weighting of hard samples ignores the contribution of the majority of low-weight easy samples in this category in the model training. Overall, Slide Loss proves to be effective in improving the recognition accuracy of hard samples.

### 4.6. Evaluation of the Proposed Model

#### 4.6.1. Ablation Experiments

To analyze the contribution of each improvement strategy to enhancing the model performance, this study conducted ablation experiments to evaluate the effectiveness of the NAM, the AFF module, the IBR module, and the Slide Loss function. The results of the ablation experiment are shown in Table 5. Firstly, YOLOv8n, used as the baseline model for this experiment, achieved a good performance of 91.7% P, 86.8% R, and 92.3% mAP at a detection speed of 95 FPS on the HGI30 dataset. Secondly, the NAM, AFF module, IBR module, and Slide Loss function were individually introduced on the basis of YOLOv8n for qualitative analysis. The mAP values achieved by each improvement strategy reached 92.9%, 93.2%, 92.7%, and 92.7%, respectively. Their Params and FLOPs were basically comparable to those of YOLOv8n. This indicates that the adopted improvement strategies can enhance model performance without significantly increasing computational costs. It is noteworthy that the NAM and the AFF form a shared module termed the Enhanced Attention Mechanism Module. Next, to demonstrate the good generalization between the improvement strategies, pairwise combinations of these strategies were performed. The experimental results show that the combination of the Enhanced Attention Module with IBR and the combination of IBR with Slide Loss achieve a 93.4% mAP and 92.9% mAP, respectively, surpassing the individual use of the corresponding improvement strategies. Finally, by combining all the improvement strategies, the proposed HGCS-Det model achieves an excellent detection performance with 92.6% P, 87.8% R, and 93.6% mAP, which is 0.9%, 1.0%, and 1.3% higher than YOLOv8n, while maintaining the low computational costs of YOLOv8n. Although the detection speed is reduced to 86 FPS due to the impact of matrix multiplication in the NAM, it still meets the real-time requirement for garbage detection.

#### 4.6.2. Qualitative Analysis

**Figure 11:**
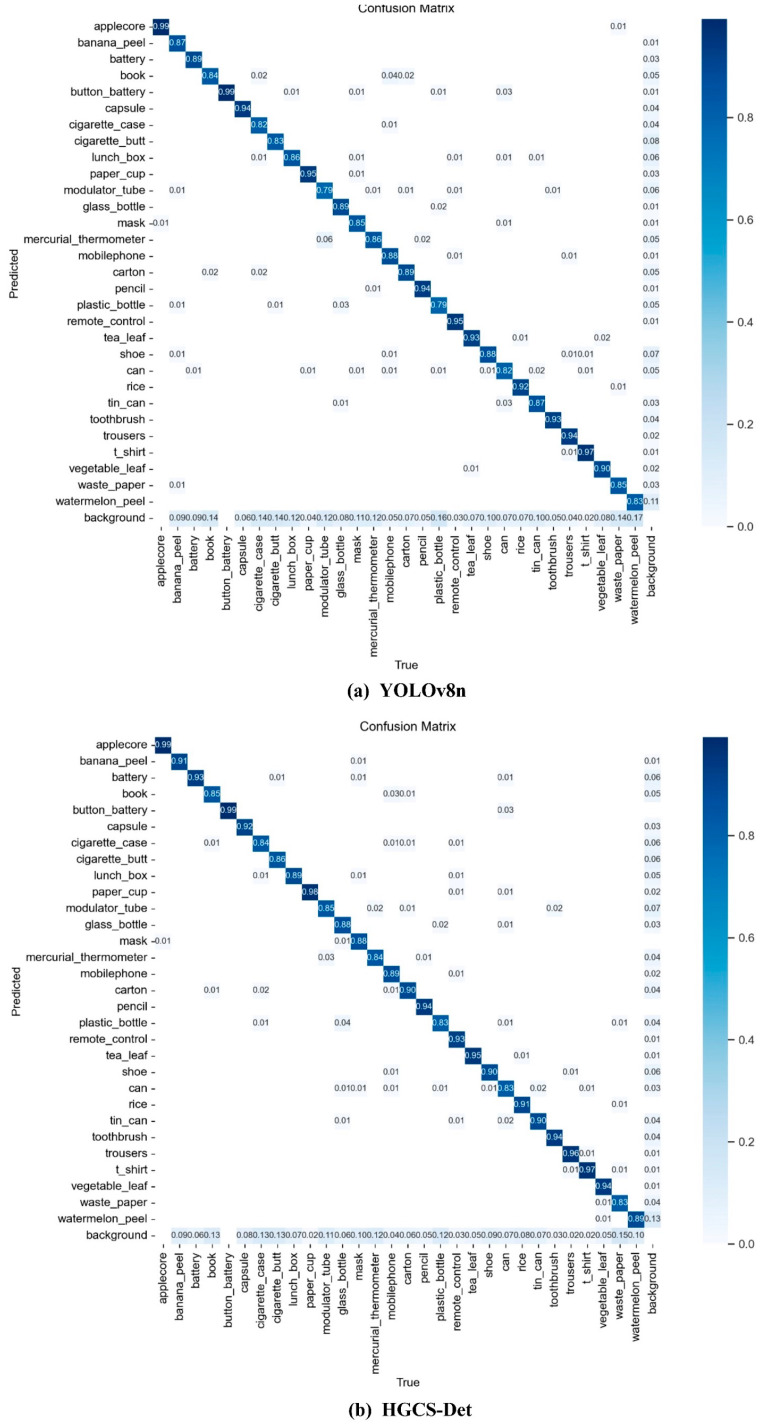
The confusion matrix results for (**a**) YOLOv8n and (**b**) HGCS-Det.

The image displays the confusion matrix results for YOLOv8n and HGCS-Det (Figure 11). The confusion matrix illustrates the recognition rates between predicted category samples and true category samples, with darker colors indicating higher recognition rates and vice versa. As illustrated in Figure 10, in YOLOv8n, the recognition accuracy for 12 classes of garbage exceeds 90%, while for 18 classes, the accuracy is below 90%, with two classes even having an accuracy of below 80%. In contrast, in HGCS-Det, the recognition accuracy for 30 classes of garbage exceeds 80%, with 17 classes having a recognition accuracy greater than 90%. Most garbage categories show an improvement in recognition rates. Additionally, HGCS-Det exhibits fewer instances of category misidentification compared with YOLOv8n. However, both models are influenced by complex scenes, with a considerable portion of category samples being misclassified as background, and a portion of backgrounds being mistakenly classified as category samples. Overall, HGCS-Det exhibits a lower proportion of these two scenarios compared with YOLOv8n, resulting in a smaller actual probability of missing detections. Hence, HGCS-Det demonstrates a slightly better performance in resisting environmental interference. Nonetheless, it is undeniable that detection tasks in complex scenes remain challenging.

### 4.7. Model Performance Comparison

#### 4.7.1. Comparison of Results with Previous Work

To validate the excellent performance achieved by HGCS-Det model on the HGI30 dataset, we conducted a comparison with results found in the literature [26]. The comparative results are presented in the table (Table 6). Previous work partitioned the HGI30 dataset into training and testing sets at an 8:2 ratio and evaluated it using six classical object detection models. Among these models, YOLOv4, leveraging the advantages of the cross-stage fusion structure for learning features, achieved a commendable detection performance with a 79.1% mAP. Furthermore, recent models such as LWCNet and MS-YOLO have made notable progress, achieving mAP values of 91.5% and 93.2%, respectively, by introducing attention-based heads, lightweight convolution modules, and multi-scale feature enhancement techniques. These improvements have significantly closed the performance gap between lightweight and heavy detection networks on complex garbage classification tasks. In contrast, employing the same data division strategy, HGCS-Det attained the highest mAP value of 93.6%, representing a significant improvement of 14.5% over YOLOv4. This marks a breakthrough achievement building upon prior research efforts.

#### 4.7.2. Comparison with Mainstream Models

**Figure 12:**
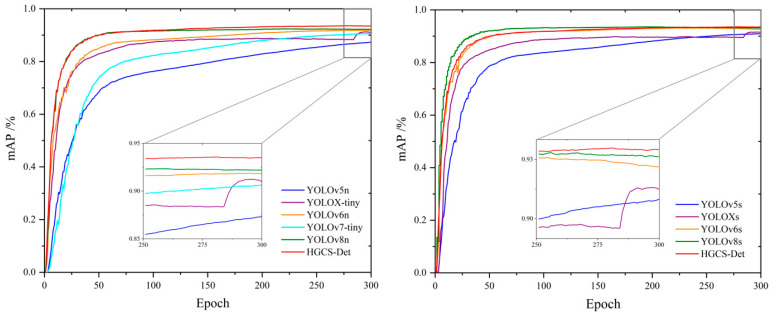
Training results of HGCS-Det with that of other mainstream models on HGI30.

To validate the superiority of the HGCS-Det model and considering that the garbage detection task requires accuracy and real-time performance, we evaluated the performances of the lightweight and sub-lightweight versions of other mainstream detection models (YOLOv5 [28], YOLOX [42], YOLOv6 [43], YOLOv7 [29], YOLOv8 [27], and YOLOv12 [44]) on the HGI30 dataset and compared these with the performance of HGCS-Det. From Figure 12, it is evident that HGCS-Det’s mAP curve rises steadily and rapidly converges until it converges to a fit, and the final mAP value obtained is higher than that of the other models. Furthermore, a detailed evaluation is shown in Table 7. The original baseline model, YOLOv8n, achieves the highest mAP among all the lightweight models with a lower volume, even surpassing the sub-lightweight models YOLOv5s and YOLOXs. Its detection speed also ranks among the highest. Moreover, the sub-lightweight model YOLOv8s also obtains the best detection accuracy among all the models, but its detection speed appears to be slightly inferior due to increased computational costs. The proposed HGCS-Det model achieves the same highest detection accuracy as YOLOv8s, with a model volume only 27% of that of YOLOv8s, which is on par with YOLOv8n. Although the detection speed is affected by the attention mechanism, it outperforms the sub-lightweight models and is even faster than the lightweight models YOLOX-tiny and YOLOv6n. Additionally, recent lightweight architectures such as LWCNet have demonstrated how a self-attention detection head (SAHead), GSConv-based GRCSPELAN blocks with GELAN, and an AIFI module can substantially reduce the parameter count while maintaining high inference speed and accuracy. Similarly, a mobile-cart-deployed variant of YOLOv8n leverages the C2f-MS backbone, a Convergence–Expansion Pyramid Network (CEPN), and a QS-Dot-IoU hybrid loss to excel at small-object detection in dynamic outdoor settings. In conclusion, HGCS-Det’s comprehensive performance is superior to that of other models, and its model size is suitable for deployment on resource-constrained devices. This indicates that HGCS-Det is more advantageous in terms of efficiency and applicability.

## 5. Discussion

This work demonstrates that integrating multi-scale feature fusion with a focused attention mechanism can enhance both precision and robustness in unstructured environments without substantially increasing model complexity. The multi-scale module captures contextual cues across resolutions, improving the detection of small or partially occluded items, while the enhanced attention block helps to suppress background noise and highlight relevant object details.

Despite these advances, HGCS-Det still incurs additional computation from the attention operations, which may impact real-time performance on highly constrained hardware. Moreover, challenging cases—such as visually similar materials under poor illumination or rapid motion—remain prone to misclassification, indicating room for further refinement.

Future efforts will concentrate on reducing the model’s computational footprint through targeted compression or pruning, as well as incorporating temporal cues to stabilize detection in video streams. We also plan to explore domain-adaptation techniques and semi-supervised learning to extend its applicability across varied waste types and deployment scenarios. These directions aim to solidify HGCS-Det’s practical utility in smart sorting systems and mobile applications.

## 6. Conclusions

This paper introduces an efficient detection model, HGCS-Det, that is designed for garbage detection in complex environments. The model mainly addresses the challenges arising from complex environmental factors and irregular garbage features that lead to the inefficiency of current detection models. Firstly, to mitigate the issue of reduced target focus caused by complex environmental conditions, the Enhanced Attention Module is proposed. In this module, an attention mechanism is paired with an attention fusion module to synthesize the weight contribution of each channel’s attention feature. This enables the model to fully utilize attention to focus on the essential information from targets and to suppress noise from the environment. Secondly, to improve the model’s capability to discriminate irregular garbage features, the Instance Boundary Reinforcement module is designed. This module integrates the gradient cues of shared convolution into difference convolution surroundings to capture target boundary features more effectively. Lastly, to address the imbalance between hard and easy samples, Slide Loss is employed to allocate higher learning weights to hard samples. This ensures that the model pays more attention during training, leading to more accurate outputs. Extensive experiments demonstrate that the proposed method exhibits strong generalization and robustness, efficiently completing garbage detection tasks in complex scenarios, and it is of great practical application significance.

However, HGCS-Det is affected by the complex matrix multiplication in the attention mechanism. In terms of running speed, it falls slightly short compared with some lightweight models, such as YOLOv7-tiny. Therefore, our future work will focus on model compression algorithms to enhance the applicability of HGCS-Det. Furthermore, we plan to leverage HGCS-Det to design mobile applications or automatic garbage classification robots from both software and hardware perspectives, enhancing its utility in garbage classification management. Additionally, we aim to explore more efficient garbage detection methods with the hope of extending the proposed approach to other object detection tasks.

## Figures and Tables

**Table 1:** Results of a comparison between attention modules and enhanced attention modules.

Model	Params/M	FLOPs/G	mAP/%	FPS
YOLOv8n	3.02	8.2	92.3	95
+SE	3.03	8.2	92.7	85
+CBAM	3.03	8.3	92.8	78
+ECA	3.02	8.2	92.7	87
+CA	3.03	8.3	93.2	81
+NAM	3.02	8.2	92.7	85
+NAM^S*^	3.02	8.2	92.6	86
+NAM^C*^	3.02	8.2	92.9	88
+SE (+AFF)	3.03	8.2	93.0 **_+0.3_**	83
+CBAM (+AFF)	3.03	8.3	93.2 **_+0.4_**	75
+ECA (+AFF)	3.02	8.2	93.1 **_+0.4_**	84
+CA (+AFF)	3.03	8.3	93.3 **_+0.1_**	80
+NAM^C*^ (+AFF)	3.02	8.2	93.2 **_+0.3_**	86

**Table 2:** Experiments on the improvement of the IBR module.

Model	Params/M	FLOPs/G	mAP/%	FPS
YOLOv8n	3.02	8.2	92.3	95
+IBE	2.60	7.2	92.0	101
+IBE (replace DWC)	3.07	8.3	92.8	87
+IBE (replace DWC and remove PC)	3.02	8.2	92.7	93

**Table 3:** Comparison results between Slide Loss and other loss functions.

Model	mAP/%
YOLOv8n	92.3
+Focal Loss	87.2
+QFocal Loss	87.4
+Varifocal Loss	88.7
**+Slide Loss**	92.7

**Table 4:** Effectiveness analysis of Slide Loss.

Category	AP/%	
	YOLOv8n	YOLOv8n + Slide
battery	86.6	92.8
cigarette case	88.3	87.8
modulator tube	84.3	84.7
watermelon peel	87.6	87.4
mercury thermometer	84.5	86.9
plastic bottle	84.7	86.2
can	85.9	88.7
waste paper	89.4	90.7

**Table 5:** Ablation experiments.

YOLOv8	NAM	AFF	IBR	SlideLoss	Params/M	FLOPs/G	P/%	R/%	mAP/%	FPS
*√*					3.02	8.2	91.7	86.8	92.3	95
*√*	*√*				3.02	8.2	92.5	86.4	92.9	88
*√*	*√*	*√*			3.02	8.2	92.1	87.8	93.2	86
*√*			*√*		3.02	8.2	92.0	86.7	92.7	92
*√*				*√*	3.02	8.2	91.2	87.9	92.7	93
*√*	*√*	*√*	*√*		3.02	8.2	92.3	87.5	93.4	83
*√*			*√*	*√*	3.02	8.2	92.0	87.3	92.9	92
*√*	*√*	*√*	*√*	*√*	3.02	8.2	92.6	87.8	93.6	86

**Table 6:** Comparison of results with previous work realized on HGI30.

Model	Data Division	Input Size	mAP/%
Faster-RCNN	8:2	1000 × 800	74.8
SSD	8:2	512 × 512	73.5
YOLOv3	8:2	608 × 608	74.3
M2Det	8:2	512 × 512	76.2
EfficientDet	8.2	512 × 512	77.6
YOLOv4	8:2	608 × 608	79.1
LWCNet	8:2	640 × 640	91.5
MS-YOLO	8:2	640 × 640	93.2
HGCS-Det	8:2	640 × 640	93.6

**Table 7:** Performance comparison of HGCS-Det with other mainstream models.

Model	Params/M	FLOPs/G	mAP/%	FPS
YOLOv5n	1.80	4.3	87.3	102
YOLOX-tiny	5.04	15.3	91.2	82
YOLOv6n	4.31	11.1	91.8	85
YOLOv7-tiny	6.09	13.4	90.6	98
YOLOv8n	3.02	8.2	92.3	95
YOLOv12n	2.6	6.5	90.3	94
LWCNet	1.7	4.3	91.5	-
MS-YOLO	2.1	6.3	93.2	-
YOLOv5s	7.10	16.2	90.9	84
YOLOXs	8.95	26.8	91.6	68
YOLOv6s	17.20	44.1	93.0	74
YOLOv8s	11.15	28.7	93.6	77
YOLOv12s	9.3	21.4	91.04	82
HGCS-Det	3.02	8.2	93.6	86

## Data Availability

The dataset analyzed in the current study is available from the research data knowledge base Zenodo (https://zenodo.org/records/4646699 accessed on 13 March 2021). Data will be made available on request.
